# Processing of Pineapple Leaf Fibers for the Production of Oxidized Micro-/Nanofibrillated Cellulose

**DOI:** 10.3390/polym17192671

**Published:** 2025-10-02

**Authors:** Marianelly Esquivel-Alfaro, Belkis Sulbarán-Rangel, Oscar Rojas-Carrillo, Jingqian Chen, Laria Rodríguez-Quesada, Giovanni Sáenz-Arce, Orlando J. Rojas

**Affiliations:** 1 Polymer Science and Technology Laboratory (POLIUNA), Department of Chemistry, Universidad Nacional, Heredia 40101, Costa Rica; oscar.rojas.carrillo@una.ac.cr; 2 Doctorado en Ciencias Naturales para el Desarrollo (DOCINADE), Instituto Tecnológico de Costa Rica, Universidad Nacional, Universidad Estatal a Distancia, Costa Rica; 3 Bioproducts Institute, Department of Chemical & Biological Engineering, The University of British Columbia, 2360 East Mall, Vancouver, BC V6T 1Z3, Canada; jchen@chbe.ubc.ca; 4 Department of Water and Energy, University of Guadalajara Campus Tonalá, Tonalá 45425, Mexico; belkis.sulbaran@academicos.udg.mx; 5 Departamento de Física, Facultad de Ciencias Exactas y Naturales, Universidad Nacional, Heredia 40101, Costa Rica; laria.rodriguez.quesada@una.cr (L.R.-Q.); gsaenz@una.ac.cr (G.S.-A.); 6 Department of Chemistry, The University of British Columbia, 2036 Main Mall, Vancouver, BC V6T 1Z1, Canada; 7 Department of Wood Science, Faculty of Forestry, The University of British Columbia, 2424 Main Mall, Vancouver, BC V6T 1Z4, Canada

**Keywords:** pineapple leaf fibers, organosolv pulping, hydrogen peroxide bleaching, lignocellulosic fibers, TEMPO-oxidized micro-/nanofibrillated cellulose

## Abstract

Pineapple leaf fibers (PALFs), obtained from abundant yet underutilized pineapple leaf residues, represent a promising feedstock for producing fibrillated cellulose. In this work, cellulosic fibers were isolated and characterized by Fiber Quality Analysis (FQA), showing lengths between 0.33 and 0.47 mm and widths of 12.2 µm after organosolv pulping using ethanol and acetic acid as a catalyst, followed by hydrogen peroxide bleaching with diethylenetriaminepentaacetic acid as a chelating agent. The cellulosic fibers were then subjected to TEMPO-mediated oxidation and subsequently disintegrated by microfluidization to produce micro-/nanofibrillated cellulose (MNFC) with a carboxylate content of 0.85 and 1.00 mmol COO^−^/g, zeta potential of −41 and −53 mV, and average widths of 15 and 12 nm for unbleached and bleached nanofibrils, respectively. The nanofibrillation yields were 73% and 68% for the bleached and unbleached MNFC samples, indicating the presence of some non-fibrillated or partially fibrillated fractions. X-ray diffraction analysis confirmed preservation of cellulose type I crystalline structure, with increased crystallinity, reaching 85% in the bleached MNFC. These findings demonstrate the feasibility of a sequential process, combining organosolv pulping, hydrogen peroxide bleaching, TEMPO-mediated oxidation, and microfluidization, for preparing MNFC from pineapple leaf fibers. Overall, this study highlights pineapple leaf residues as a sustainable source of MNFC, supporting strategies to transform agricultural waste into valuable bio-based materials.

## 1. Introduction

Pineapple harvesting and industrial processing produce large amounts of lignocellulosic residues, including leaves, stems, peels, and crowns [1]. Among these, pineapple leaf waste is particularly rich in cellulose and represents a promising feedstock for multiple value-added applications, including natural fiber production for textiles and paper, bioethanol and biogas generation, composting, biochar production, and the recovery of bioactive compounds [2,3,4].

Biomass processing generally involves the fractionation of cellulose, hemicellulose, and lignin through physical, chemical, thermal, biological, or combined pretreatments. These treatments are designed for the extraction of cellulosic fibers for papermaking, regardless of the loss and degradation of byproducts such as lignin, hemicellulose, and extractables [5].

The most common pulping process for pineapple leaf fibers is alkaline pulping, using sodium hydroxide (2 to 8%), temperatures from 25 °C to 80 °C, and times from 1 to 24 h [6,7,8,9]. Steam explosion coupled with alkaline treatment has also been reported [10,11]. These pulping processes are usually followed by bleaching with sodium hypochlorite or sodium chlorite [6,9]. In some cases, however, bleaching is omitted depending on the process applied to obtain nanocellulose [7]. The need to develop chlorine-free alternatives, such as hydrogen peroxide bleaching, has also been highlighted in the literature due to concerns about environmental risks [12].

Recent efforts have considered clean and sustainable pulping technologies consistent with green chemistry principles. One such alternative is organosolv pulping, which uses organic solvents to selectively extract lignocellulosic components. This method is drawing attention because of its efficiency in separating cellulose from hemicellulose and lignin and is compatible with sustainable processing goals [13,14]. Conventional pulping and bleaching generate secondary pollutants that pose environmental concerns [7,8]. In contrast, organosolv pulping represents a milder alternative with a reduced chemical load. The process offers the advantage of solvent recovery by distillation due to the use of a low-boiling solvent, and the potential recovery of byproducts from the cooking liquor [15].

For this purpose several organic solvents, including alcohols like ethanol or methanol; organic acids such as acetic acid or formic acid; acetone; peroxides; and ketones have been employed alone or in combination with water and mineral acids to facilitate delignification [16,17,18]. In a large variety of lignocellulosic feedstocks, organosolv pulping has been reported; these include hardwoods and softwoods [19], agricultural or agro-industrial residues like corn cobs [20,21], sugarcane bagasse [22], agave bagasse [23], and bamboo [24], among others.

Several organosolv processes have reached pilot or demonstration scale, some examples include the Lignol process (Canada) and the Fraunhofer Centre process (Germany), both based on ethanol and producing ethanol, lignin, and xylose. The Glycell process (Australia) uses glycerol/H_2_SO_4_, and the CIMV company process (France) is based on acetic acid/formic acid. The Formico process (Finland) applies formic acid; a plant is under development and expected to be operational by 2027 [5].

Although these reports illustrate the technical feasibility of the organosolv process, the application to pineapple residues remains very limited. Few studies have reported on organosolv processes for extracting cellulosic fibers from pineapple plants. Studies on pineapple leaves include the use of acetone to obtain cellulosic fibers for paper production [25] and acetic acid/hydrochloric acid, focused on paper sheets preparation [26]. There is also organosolv pulping of pineapple crown with acetic acid and hydrochloric acid as a catalyst [27] and pretreatment with diluted sulfuric acid and hydrochloric acid (HCl), followed by organosolv pulping with ethanol, for pineapple peel for the generation of fermentable sugars for bioethanol production [28].

However, to the best of our knowledge, application of ethanol-based organosolv pulping to pineapple leaf fibers as a precursor process for nanocellulose production has not yet been reported. In this context, the production of micro-/nanofibrillated cellulose (MNFC) represents a sustainable pathway for the valorization of pineapple leaf fibers. Cellulose nanofibers are elongated structures with widths of nanometric size and micrometric length; they combine crystalline and less ordered regions that enable network formation. Several techniques are applied to extract nanofibers, like mechanical techniques, such as high-pressure homogenization and ultrasonic treatment; these approaches are milder than acid hydrolysis, which is most often used to produce cellulose nanocrystals [29].

One way to facilitate mechanical fibrillation is the chemical modification of cellulose by incorporation of ionic charges. An example of this modification is (2,2,6,6-tetramethylpiperidinyloxy) TEMPO-mediated oxidation that incorporates carboxylate groups on the cellulose surface [30]. There are a few previous studies on the use of this strategy to treat pineapple leaf biomass [6,9,31].

Hence, the present study proposes the production of micro-/nanofibrillated cellulose (MNFC). The nanomaterial is produced by applying a sequential ethanol-based process with optional hydrogen peroxide bleaching, followed by TEMPO oxidation and subsequent mechanical processing by microfluidization.

## 2. Materials and Methods

### 2.1. Materials

Ethanol (C_2_H_6_O, ≥99.8%), acetic acid (CH_3_COOH, ≥99.7%), hydrogen peroxide (H_2_O_2_, 30%), diethylenetriaminepentaacetic acid (DTPA, C_14_H_23_N_3_O_10_, ≥98%), TEMPO (2,2,6,6-tetramethylpiperidine-1-oxyl, C_9_H_18_NO, 98%), sodium hypochlorite (NaClO, 10–15% available chlorine), sodium bromide (NaBr, ≥99.0%), hydrochloric acid (HCl, 37%), sodium hydroxide (NaOH, ≥97.0%), and calcofluor white dye. All the chemical reagents were purchased from Sigma-Aldrich (Saint Louis, MO, USA). The chemicals used were analytical grade, and they were used as received without further purification.

### 2.2. PALF Extraction and Preprocessing

**Figure 1:**
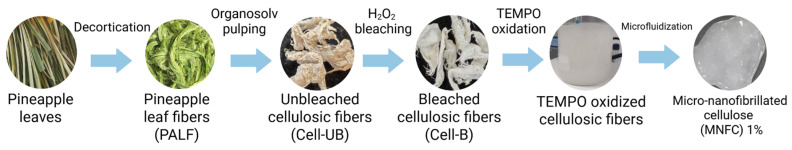
Overview of the sequential process for converting pineapple leaf fibers into micro-/nanofibrillated cellulose (MNFC) (created in BioRender.com/bvw26z2).

The overall workflow for converting pineapple leaf fibers into TEMPO-oxidized micro-/nanofibrillated cellulose (MNFC) is shown schematically (Figure 1). Pineapple leaves were collected from the Huetar Norte region of Costa Rica and supplied by the Eco Sweet company. All the experiments were carried out on a single large batch of pineapple leaves. The leaves were decorticated using a Reinartz (Neuss, Germany) decortication machine at the Universidad Técnica Nacional, Costa Rica (San Carlos Campus). The machine features a scraped roller that peels the cuticle and non-fibrous tissues and collects the fibrous fraction, yielding pineapple leaf fibers (PALFs). The by-product of this process (non-fibrous fraction) is used for other research projects. The extracted pineapple leaf fibers were dried in a convection oven at 60 °C.

### 2.3. Organosolv Pulping and Optional Bleaching

To isolate cellulosic fibers from pineapple leaf fibers (PALF), non-cellulosic components, including lignin and hemicellulose, must be selectively removed. A total of 400 g of dried PALF was subjected to organosolv pulping in a Jaime-type digester, a custom-built (non-commercial) laboratory-scale reactor, the solvent used was ethanol, and the mixture was prepared by adding 2 L of water, 2 L of absolute ethanol, and 12 mL of acetic acid. The pulping temperature used was 175 °C, and the time applied was 2.5 h, following the methodology described by Hernández, Romero, Escalante, Toriz, Rojas, and Sulbarán-Rangel [23]. The organosolv liquor was collected for subsequent recovery of lignin; its characterization is part of a separate study. Some of the material was bleached using 50 g of wet fibers with a solution of hydrogen peroxide (30%), 1:1 (*v/v*), and deionized water (600 mL total volume). A total of 10 mL of diethylenetriaminepentaacetic acid (DTPA) was added as a chelating agent. The reaction was performed at 95 °C for 75 min under continuous agitation at pH 11.

### 2.4. Pineapple Leaf and Fiber Morphology Analyses

Confocal fluorescence images of transversal pineapple leaf sections were acquired using the Evident SpinSR spinning disk system, operated with the cellSens Dimension software version 4.1. Images were obtained with a Yokogawa CSU-W1 (50 µm disk) and 20× NA 0.8 Plan Extended Apochromat objective lens. Multi-channel Z-stack (44 slices at 3 µm steps) images were captured in a sequential fashion (channel priority) with on-the-fly tiling and stitching. Fluorescence signals were obtained with a Hamamatsu ORCA Fusion BT sCMOS camera. Excitation/emission filter sets included 405/447–60 nm, 488/525–50 nm, and 561/617–73 nm. The PALF samples were analyzed using a confocal/multiphoton microscope (Stellaris 8 DIVE, Leica Microsystems, Wetzlar, Germany), and fibers were stained with calcofluor white (1 g/L) to visualize the cellulose. The images were collected sequentially using excitation wavelengths of 405 nm (emission: 447/60 nm) for calcofluor-stained cellulose and 488 nm (emission: 525/50 nm) to capture autofluorescence from lignin.

The fiber morphology of the extracted cellulosic fiber samples was assessed using a Fiber Quality Analyzer (FQA) (OpTest Equipment Inc., Hawkesbury, ON, Canada), in accordance with ISO 16065 [32]. Fiber suspensions were prepared in deionized water and dispersed using magnetic stirring, followed by pulp homogenization. Morphological parameters measured by FQA, including fiber length, fiber width, percentage of fines, curl, and kink indexes, were measured for approximately 5000 fibers per sample, in duplicate, and average values were reported. The morphology of PALFs and the extracted cellulosic fibers were also observed by scanning electron microscopy (SEM). The samples were dried at 60 °C for 24 h, mounted on standard holders with double-sided carbon tape, and coated with a gold layer with a thickness of approximately 15 nm in an ion coater (G20, EMCRAFTS, Hanam-si, Republic of Korea), at 10 mA for 120 s. SEM images were acquired with a Cube-II (EMCRAFTS, Republic of Korea) scanning electron microscope under high vacuum conditions (7.5 × 10^−5^ Torr) at 10–15 kV.

### 2.5. Micro-/Nanofibrillated Cellulose (MNFC)

To obtain MNFC (TEMPO-oxidized micro-/nanofibrillated cellulose) from pineapple leaf fibers, a sequential process involving chemical pretreatment followed by microfluidization was employed. The chemical modification step involved TEMPO-mediated oxidation, for unbleached and bleached cellulosic fibers, using Saito et al.’s [33] methodology. This process is known for the selective oxidation of the primary hydroxyl groups located at the C_6_ position of the glucose monomer in the cellulose backbone, producing carboxylate groups. The oxidation was carried out at 25 °C and pH 10.5 (using NaOH 0.1 M), 2.5 mmol of sodium hypochlorite (NaClO) per gram of dry fibers was used for this oxidation. The TEMPO-oxidized cellulose was washed and dialyzed, and then it was suspended in water to reach 1 wt% solids, and the suspension was subsequently disintegrated mechanically using a high-pressure homogenizer (LM10 Microfluidizer, Westwood, MA, USA) equipped with a 100 µm Z-type interaction chamber. This device generates intense shear and impact stresses capable of breaking down the oxidized cellulose into nanoscale fibrils. Two passes were applied at 8000 kPa, yielding a viscous MNFC hydrogel. The nanofibrillation yield was determined in triplicate, following the procedure reported by Aguado et al. [34]. Briefly, a suspension (0.2 wt%) was centrifuged at 4500 rpm for 20 min (Eppendorf 5804R, Hamburg, Germany) and the sediment was considered as the non-fibrillated or partially fibrillated fraction, with the supernatant assigned to the nanofibrillated fraction. The sediment was oven-dried, and the yield was calculated according to the following equation:

(1) $$Nanofibrillation\;yield\;\left(\%\right)=\;\frac{Dry\;weight\;of\;supernatant\;}{Dry\;weight\;of\;centrifuged\;sample}\;\times 100$$

### 2.6. Characterization

A sequential characterization strategy was applied to evaluate the morphological and structural evolution of pineapple leaf fibers (PALF) throughout processing. FQA and SEM were used to assess the precursor cellulosic fibers at the micrometer scale, as explained in Section 2.4, whereas transmission electron microscopy (TEM) and atomic force microscopy (AFM) provided nanoscale information of the final MNFCs. These analyses were complemented by zeta potential, X-ray diffraction (XRD), Fourier-transform infrared spectroscopy (FTIR), conductimetric titration, and thermogravimetric analysis (TGA) to assess structural, chemical, and thermal properties. The following subsections describe each technique in detail, including the conditions and procedures applied.

Zeta Potential: The zeta potential of MNFC was quantified with a Nanosizer (ZS90, Malvern Instruments, Worcestershire, UK). The samples were prepared by dispersion of the microfluidized hydrogel in deionized water at pH 7. A total of 15 scans per measurement were repeated three times; the values reported are average values in millivolts (mV).

MNFC morphology: TEM images were obtained using Transmission Electron Microscopy (H7700, Hitachi, Tokyo, Japan) to observe the morphology of the MNFCs. For this purpose, a diluted aqueous suspension of MNFC (~0.0002%) was drop-cast (5 µL) onto 200-mesh carbon-coated copper grids and dried. Staining was performed with 0.5% (*w*/*v*) phosphotungstic acid. The samples were analyzed at 70 kV at magnifications of 10,000× to 20,000×. AFM imaging was conducted using an Atomic Force Microscope (NX10, Park Systems, Gyeonggi-do, Republic of Korea); mica holders were freshly cleaved, and a 20 µL drop of diluted microfluidized suspension was deposited and dried at room temperature. The imaging was performed in true-contact mode using Tap-300Al-G probes (Innovative Solutions Bulgaria Ltd., Sofia, Bulgaria) applying a resonance frequency of 300 kHz, a spring constant of 40 N/m, and tip radius under 10 nm. Image processing was performed using the Park Smart Analysis software (1.5.0.1990, Park Systems, Suwon, Republic of Korea) and XEI software (5.2.0.Build1, Park Systems, Suwon, Republic of Korea). Nanofiber dimensions were quantified by 50 width measurements using the ImageJ software (Version 1.54p, National Institutes of Health, Bethesda, MD, USA). For both the AFM and TEM images, mean values with standard deviations were calculated, and the corresponding width distribution was analyzed through histograms.

Crystallinity: X-ray diffraction (XRD) analysis was carried out using an Empyrean diffractometer (Siemens, Karlsruhe, Germany, USA) with CuKα radiation (λ = 1.5405 Å). Scans were recorded over a 2θ range of 5–50°. The crystallinity index (CI) of the samples was estimated using the Segal method, a widely used empirical and semi-quantitative approach based on XRD peak intensities. The method uses the intensity ratio of relevant regions of the diffractogram, according to the following equation [35]:

(2) $$CI=\frac{{(I}_{002}-I_{am})}{I_{002}}\times 100$$

where I_002_ is the maximum intensity of the refractive peak (002) at 2θ ≈ 22–23° for cellulose I, and I_am_ is the intensity corresponding to the disordered component, measured at 2θ = 18°.

Chemical analyses: Fourier-transform infrared spectroscopy (FTIR) spectra were recorded utilizing a Nicolet iS50 spectrometer (Thermo Scientific, Waltham, MA, USA) equipped with an attenuated total reflectance (ATR) accessory. Each spectrum was obtained by applying 32 scans in the wavelength range from 500 to 4000 cm^−1^. Analyses were conducted in duplicate for PALF, cellulosic fibers, and MNFCs to identify functional groups and compositional changes. The carboxyl group content of MNFCs was quantified via conductometric titration following the Saito and Isogai [36] method. Freeze-dried samples were dispersed in deionized water, the pH was adjusted with HCl to reach 2.5, and titration was conducted with 0.05 M NaOH under continuous conductivity measurement using a conductivity meter (Orion 3 Star, Thermo Scientific, USA). Equation (2) was used to determine the carboxyl content (mmol COO^−^/g fiber):

(3) $$Carboxylate\;content=\frac{\left(V_{2}-V_{1}\right)\;\times \;C_{NaOH}}{m}$$

where V_2_ and V_1_ are NaOH volumes consumed during the titration, C_NaOH_ is the sodium hydroxide molar concentration, and m is the dry mass of the sample analyzed. The titrations were repeated three times per sample, and average values are reported.

Thermal analysis: Thermal stability was assessed using a thermogravimetric analyzer (TGA Q500, TA Instruments, New Castle, DE, USA). The samples (PALF, cellulosic fibers, and MNFCs) were analyzed by heating from 25 °C to 650 °C at a constant heating rate of 20 °C/min, using a nitrogen atmosphere with a flow rate of 40 mL/min. All the analyses were performed in duplicate.

## 3. Results and Discussion

### 3.1. Organosolv Pulping and Hydrogen Peroxide Bleaching

**Figure 2:**
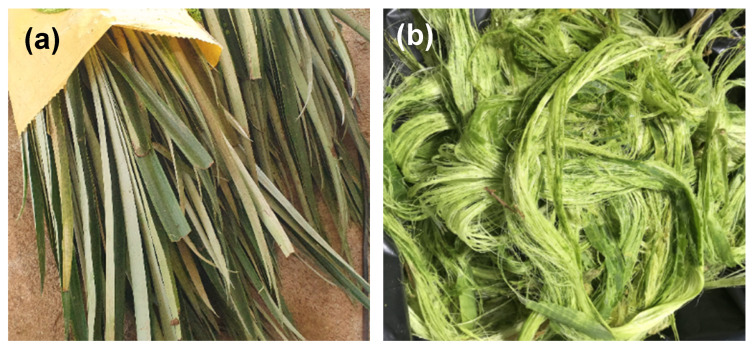
Photographs of (**a**) raw pineapple leaves and (**b**) pineapple leaf fibers (PALF) obtained through mechanical decortication.

Figure 2a shows the raw pineapple leaves, and Figure 2b the fibers obtained through mechanical decortication, referred to as pineapple leaf fibers (PALFs). The decortication process enabled the separation of fibrous from non-fibrous tissues, thereby improving the efficiency of subsequent cellulosic fibers extraction [37,38]. To investigate the structural features of the raw and processed materials, confocal fluorescence microscopy was employed to understand the microstructural organization of the fibers.

**Figure 3:**
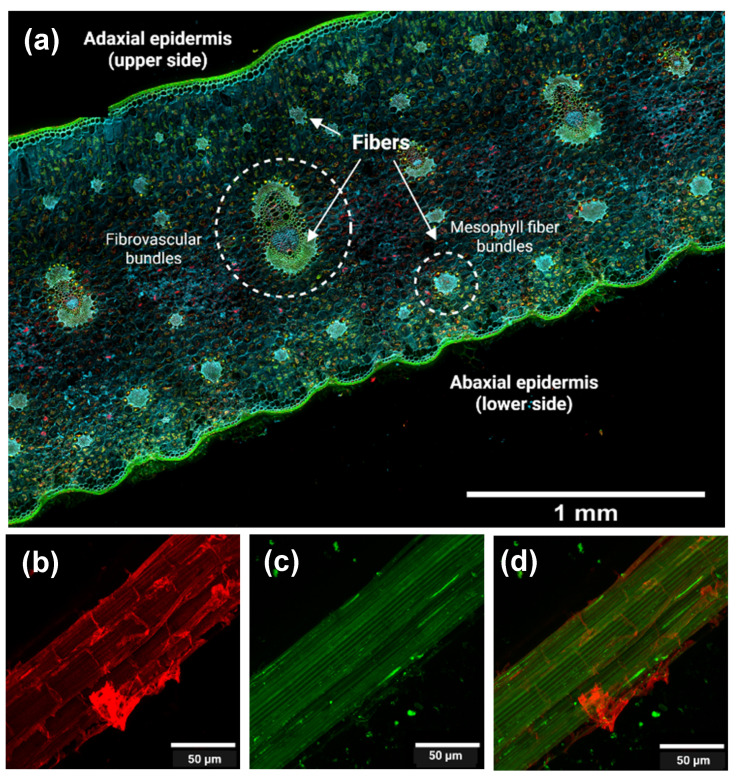
(**a**) Confocal fluorescence microscopy of pineapple leaf (transversal cut). Confocal fluorescence microscopy of PALF (pineapple leaf fibers) obtained after decortication: (**b**) cellulose (calcofluor white-stained), (**c**) lignin (autofluorescence), and (**d**) merged image. The imaging takes advantage of the autofluorescence of lignin and the fluorescence of calcofluor white, a dye that specifically binds to cellulose.

The transverse section of the pineapple leaf (Figure 3a) reveals both the adaxial epidermis, which is the upper surface of the leaf, and the undulated abaxial epidermis corresponding to the lower surface of the leaf. Fibrous tissues are located in fibrovascular and mesophyll fiber bundles, distributed across the leaf. This fiber distribution is consistent with that described by Todkar and Patil [39]. During decortication, only the fibrous elements were retained, while surrounding parenchymal and epidermal tissues were removed mechanically.

Figure 3b,c correspond to confocal microscopy images of PALF at the microscale. These images highlight cellulose (Figure 3b), lignin (Figure 3c), and both components in the merged image (Figure 3d) to assess the component distributions after decortication.

**Figure 4:**
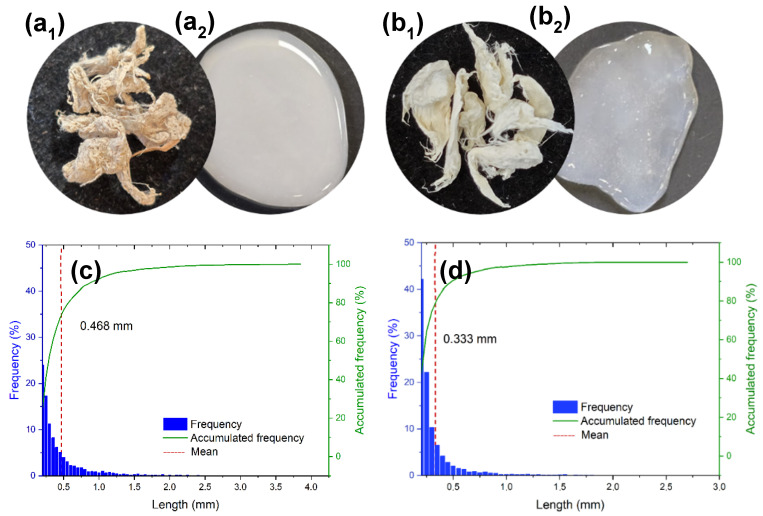
Cellulosic fibers extracted from PALF: (**a_1_**) unbleached (Cell-UB) and (**b_1_**) bleached (Cell-B). The corresponding fiber length distribution and frequency for (**c**) unbleached and (**d**) bleached fibers are also included. After TEMPO-oxidation and microfluidization, the unbleached and bleached fibers yielded micro-/nanofibrillated cellulose (MNFC) that formed hydrogels with water: (**a_2_**) MNFC-UB, and (**b_2_**) MNFC-B, respectively.

In Figure 3b, cellulose appears as bundles that provide structural support to the cells, and the microfibrils are surrounded by the other components (lignin and hemicellulose). In Figure 3c, lignin appears colocalized with cellulose, supporting its role as a structural “glue” that enhances rigidity and cohesion, and provides evidence for the separation of fibrous and non-fibrous tissues. The merged image (Figure 3d) clearly illustrates the distinct but associated distribution patterns of these components. Figure 4(a_1_,b_1_) show the cellulosic fibers extracted from PALF following organosolv pulping, both before (unbleached, Figure 4a_1_) and after (bleached, Figure 4b_1_) the bleaching treatment.

In this study, a combined organosolv system based on ethanol and acetic acid was used. Ethanol acts as the primary solvent, whereas acetic acid enhances catalytic activity by increasing proton concentration in the reaction medium. This acidification promotes hydrolytic cleavage of lignin and hemicellulose bonds, improving delignification efficiency while preserving cellulose. Moreover, the process disrupts lignin–carbohydrate complexes, thereby reducing the residual hemicellulose content [23,40].

From an environmental perspective, the organosolv method offers significant advantages. Ethanol can be easily recovered through distillation, and the lignin produced is free of sulfur compounds, making it suitable for high-value applications [41,42]. Under the conditions used for bleaching, hydrogen peroxide forms hydroperoxide anions (HOO^−^), which decompose chromophoric structures in lignin and other colored compounds, resulting in effective decolorization of cellulosic fibers [12]. To enhance bleaching efficiency and stabilize hydrogen peroxide, chelating agents such as diethylenetriaminepentaacetic acid (DTPA) are added. As a polycarboxylic acid, DTPA binds metal ions like iron (Fe^2+^), manganese (Mn^2+^), and copper (Cu^2+^) that would otherwise be able to catalyze the decomposition of hydrogen peroxide. This stabilization extends the oxidative activity of hydrogen peroxide and improves the bleaching process [43].

The morphological characteristics of the extracted cellulosic fibers, both unbleached and bleached, after organosolv pulping and bleaching were assessed using FQA and SEM analyses. FQA, designed for analyses of micrometer-scale fibers, was applied to the precursor materials to obtain quantitative parameters such as fiber length, width, and fines content. SEM, in turn, provided complementary information by directly observing fiber morphology and surface features, while also allowing diameter measurements that corroborate the micrometric size and extend the data obtained from FQA. The fiber length distribution obtained by FQA, along with the accumulated frequency data, is presented in Figure 4c,d, while the corresponding quantitative results are summarized in Table 1.

The cellulosic fibers from the unbleached material showed a broader length distribution, with fibers reaching up to approximately 4 mm in length. In contrast, the bleached cellulosic fibers exhibited a narrower length distribution, with a higher proportion of shorter fibers and with few exceeding 1.5 mm, although some fibers reach a maximum of around 3 mm. This variation is also evident from the mean fiber length, indicated by the red dashed line in the length histograms, which is lower for the bleached sample. These results reveal the impact of the bleaching process, which contributes to fiber shortening, possibly due to partial degradation and breakage induced by oxidative conditions.

The unbleached cellulosic fibers exhibited a mean fiber length of 0.47 mm, whereas the bleached cellulosic fibers had a significantly shorter mean length of 0.33 mm, indicating that the bleaching process led to an important reduction in fiber length. This chemically induced reduction in size also results in a marked increase in the proportion of fine, short fibers defined by ISO 16065 [32] as having lengths less than 0.20 mm. In contrast, the fiber width remains similar, which indicates that the transverse structure of the fibers was not significantly affected by bleaching.

Bleached cellulosic fibers exhibited a higher curl index, probably linked to fiber shortening. The kink index remained stable, showing only a slight increase, along with a small decrease in the total kink angle. However, the kinks per millimeter remained nearly constant, suggesting that the degree of fiber deformation is not substantially altered.

Although hydrogen peroxide is regarded as a mild oxidizing agent and a more sustainable alternative to chlorine-based reagents, it can still degrade cellulose. The addition of DTPA helps stabilize hydrogen peroxide by sequestering metal ions; it does not entirely prevent the formation of hydroxyl radicals, which can cause structural damage to cellulose through chain cleavage and oxidation. During bleaching with hydrogen peroxide, the formation of hydroperoxide anions (HOO^−^) occurs, and this can generate OH radicals, which can attack the β-1,4-glycosidic bonds of cellulose, thereby shortening the cellulose chains [44]. Through the process, hemicellulose dissolves and lignin is oxidized, which generates carboxylic acids and other degradation products [45]. These combined reactions compromise fiber integrity and generate structural degradation by removing amorphous components of the material [46,47].

The cellulosic fiber lengths obtained in this study are relatively shorter in comparison with other fibers. For instance, Kumar et al. [48] reported average fiber lengths of 1.8 mm for fique pulp, 1.7 mm for Northern Bleached Softwood Kraft (NBSK), and 0.61 mm for bleached eucalyptus kraft (BEK). However, for pineapple leaf fibers specifically, Ferdous [49] reported fiber lengths of 0.783 mm and a width of 11.3 µm following a Soda-AQ (anthraquinone) pulping process, values that are in close agreement with those observed in the present study.

**Figure 5:**
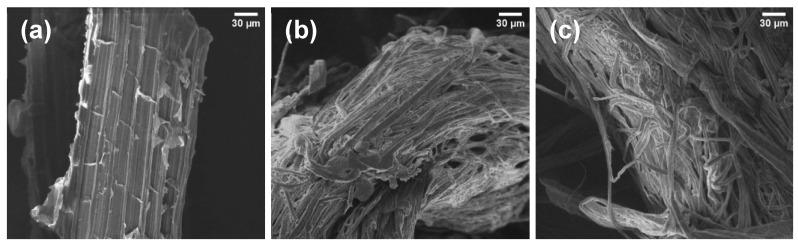
SEM images of PALF (**a**), and cellulosic fibers extracted from PALF: (**b**) unbleached (Cell-UB) and (**c**) bleached (Cell-B).

In order to compare the morphological differences between PALF and the extracted cellulosic fibers—unbleached (Cell-UB) and bleached (Cell-B)—SEM analyses were performed. In Figure 5a, the PALF structure can be observed, which is very similar to the structures observed in the confocal fluorescence microscopy analysis (Figure 3b–d), showing organized and compact fiber bundles. After organosolv pulping, the unbleached cellulosic fibers (Cell-UB, Figure 5b) exhibited a loss of the ordered structure, with partially disaggregated fibers, evidencing the effect of pulping on partial removal of components such as lignin and hemicellulose. The average width of the cellulosic fiber was 13 ± 3 µm. After the bleaching process, the bleached cellulosic fibers (Cell B, Figure 5c) showed cleaner and more homogeneous fibers, with smoother surfaces, related to the bleaching process that removes non-fibrous components more extensively, and the width was reduced to 11 ± 3 µm. These observations highlight the progressive process applied to PALF through pulping and bleaching for obtaining unbleached and bleached cellulosic fibers. Moreover, SEM images confirm that the fiber diameters remain in the micrometer range (11 to 13 µm), consistent with the FQA results (~12 µm). Thus, SEM confirms the quantitative analyses by providing direct visualization of fiber morphology and surface features, while FQA provides statistical measurements of fiber dimensions.

### 3.2. Morphology of MNFC from PALF

Following fiber extraction, TEMPO-oxidation and microfluidization were performed, yielding TEMPO-oxidized micro-/nanofibrillated cellulose (MNFC) hydrogels from unbleached cellulose (MNFC-UB, Figure 4a_2_) and bleached cellulose (MNFC-B, Figure 4b_2_). The TEMPO oxidation imparted a negative surface charge to the cellulose, enhancing its dispersibility in water, attributed to the electrostatic repulsion among the micro-/nanofibers. The cellulose oxidation by introducing carboxylate groups facilitates the subsequent mechanical processing, as the repulsive forces between negatively charged fibrils reduce interfibrillar hydrogen bonding and promote fiber separation [30,50].

MNFC samples were obtained with nanofibrillation yields of 68 ± 4% (unbleached) and 73 ± 1% (bleached), indicating that most of the material reached the nanoscale, although residual fractions remained non-fibrillated or partially fibrillated (32% for UB and 27% for B). For other lignocellulosic sources subjected to TEMPO oxidation, reported nanofibrillation yields vary widely—for example, 41.4–62.3% for old corrugated containers (OCC, 5–15 mmol NaClO/g) [51], 42.2 to >95% for bleached Aspen TMP (thermo-mechanical pulp, 5–10 mmol NaClO/g) [52] and 94.61–99.13% for bleached kraft eucalyptus pulp (BKEP, 5–25 mmol NaClO/g) after six microfluidization passes [53]. Such differences are influenced by the oxidant dosage, as well as the type and intensity of subsequent mechanical treatments.

In our study, 2.5 mmol NaClO/g and only two microfluidizer passes were applied, yet the resulting nanofibrillation yields were higher than those reported for OCC [51] and bleached Aspen TMP [52] despite using a lower oxidant dose and fewer passes. No subsequent separation of micro- and nanofibrillated fractions was performed, as the intended application of the hydrogel does not require such separation.

**Figure 6:**
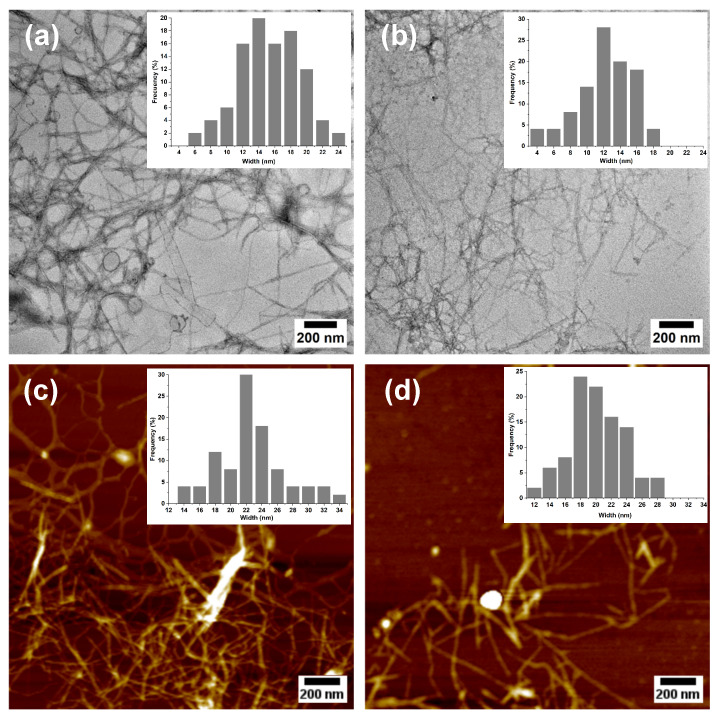
TEM (**a**,**b**) and AFM (**c**,**d**) images of micro-/nanofibrillated cellulose: MNFC-UB (unbleached, (**a**,**c**)) and MNFC-B (bleached, (**b**,**d**)) with corresponding histograms showing the width distribution.

To examine the morphology of MNFCs at the nanoscale, Transmission Electron Microscopy (TEM) was conducted (Figure 6a,b). In both samples, a network of nanofibrillar structures is evident, showing micrometer lengths and nanometer widths. The unbleached micro-/nanofibers (MNFC-UB) tend to exhibit greater agglomeration and irregularity, whereas the bleached micro-/nanofibers (MNFC-B) appear more individualized, thinner, and evenly distributed. This improved dispersion in the bleached sample is associated with its higher degree of oxidation and corresponding increase in surface charge, as will be shown later, which enhances interfiber electrostatic repulsion and facilitates separation.

Additional morphological insights were obtained using atomic force microscopy (AFM, Figure 6c,d), which revealed similar features to those observed via TEM, with both samples forming entangled networks of long and slender nanofibers. The MNFC-B displayed improved dispersion or reduced aggregation, consistent with a higher surface charge. Quantitative dimensional analyses conducted using TEM and AFM confirmed that TEMPO-oxidized micro-/nanofibrillated cellulose retained micrometer-scale lengths but exhibited widths in the nanometer range. Due to the network-forming morphology of the nanofibers, direct quantification of fibril length was not feasible. However, their widths were determined as 15 ± 4 nm (TEM) and 22 ± 4 nm (AFM) for MNFC-UB, and 12 ± 3 nm (TEM) and 20 ± 4 nm (AFM) for MNFC-B. Corresponding histograms for each image illustrate the size distribution and widths of the MNFCs.

These results demonstrate the mechanical disintegration of both the unbleached and bleached cellulosic fibers, with the slightly smaller widths in the bleached sample suggesting more efficient fibrillation, likely due to enhanced fiber swelling and charge repulsion induced by higher oxidation. Since these dimensions fall well below the 100 nm threshold, the individual fibrils can be classified as nanofibers [54]. This nanoscale evidence, combined with comparative FQA and SEM data at the micrometer level, supports the extent of fibrillation achieved for pineapple leaf fibers. It should be noted, however, that TEM and AFM require highly diluted samples and capture only small fractions of the population, and thus may not fully represent the bulk suspension.

According to the definition of ISO/TS 20477:2017 [55], the term nanofibrillated cellulose (NFC) is based on the morphological characteristics of the fibrils, and some might have functional groups derived from the manufacturing process. The nanofibrillation yields obtained correspond to suspensions where the largest proportion of material is at the nanoscale, while a residual fraction remains at the micrometric scale; for this reason and following the terminology commonly used in the literature, the obtained product is classified as micro-/nanofibrillated cellulose (MNFC).

To provide clear evidence of the progressive micro-/nanofibrillation of pineapple leaf fibers, a comparative morphological analysis was conducted at each key stage of processing. The transformation pathway was documented from raw PALF and cellulosic fibers (unbleached and bleached) characterized by SEM and FQA, to the final MNFC products analyzed by TEM and AFM. This sequential comparison highlights the progressive reduction in fiber dimensions, from micrometer-scale extracted cellulosic fibers to nanometer-scale fibrils. By integrating multiple imaging techniques, morphological evidence clearly demonstrates the conversion into micro-/nanofibrillated material.

The FQA, SEM, TEM, and AFM techniques were selected according to the characteristic scale of observation: FQA and SEM were employed to evaluate micrometer-sized cellulosic fibers, while TEM and AFM provided the nanoscale resolution required to confirm the successful production of nanofibrils. Table 2 summarizes the morphological progression from raw pineapple leaf fibers (PALFs) to TEMPO-oxidized micro-/nanofibrillated cellulose (MNFC). The results clearly illustrate the dimensional transition from millimeter and micrometer-sized cellulosic fibers to nanometer-scale structures: fiber lengths in the hundreds of micrometers were converted into networks of nanofibrils with widths below 25 nm. This comparative evidence demonstrates the effectiveness of the sequential process.

### 3.3. Chemical and Structural Features of MNFC from PALF

**Figure 7:**
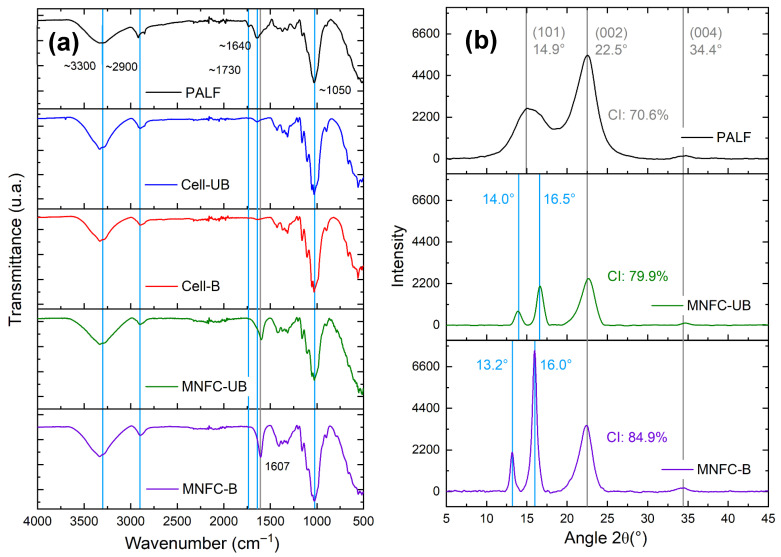
(**a**) FTIR spectra of PALF, extracted cellulosic fibers (unbleached and bleached), and TEMPO-oxidized micro-/nanofibrillated cellulose (MNFC-UB and MNFC-B). (**b**) X-ray diffraction (XRD) patterns of PALF, MNFC-UB, and MNFC-B.

The FTIR spectra for pineapple leaf fibers (PALF), extracted cellulosic fibers (unbleached and bleached), and the corresponding micro-/nanofibers (MNFC-UB and MNFC-B) are shown in Figure 7a. All the spectra exhibit the characteristic absorption bands of cellulose. These include a wide signal at 3300 cm^−1^ assigned to O–H stretching vibrations, a peak around 2900 cm^−1^ related to C–H stretching of the cellulose chain, and also a prominent peak at 1050 cm^−1^ linked to C–O–C stretching of glycosidic bonds [56]. Although chemical modifications are applied throughout the process, these spectral features remain consistent, indicating preservation of the fundamental cellulose structure, likely because the reactions occur primarily on the fiber surface.

In comparison with PALF, the spectra of the extracted cellulosic fibers reveal a noticeable intensity decrease in the band associated with the C=O stretching vibration of ester groups in hemicelluloses, near 1730 cm^−1^ [56]. This reduction is attributed to acid-catalyzed hydrolysis during the organosolv pulping, which solubilizes hemicelluloses, as previously reported for other biomass sources such as sugarcane straw [22] and agave bagasse [23].

Additionally, a reduction in absorbance around 1640 cm^−1^ is observed, a region typically associated with C=C stretching vibrations of aromatic rings in lignin [57]. The decrease confirms partial removal of lignin during pretreatment and its near-complete elimination in the final micro-/nanofiber samples.

Notably, the FTIR analysis for MNFCs exhibits a new absorption band near 1607 cm^−1^, corresponding to the asymmetric stretching of carboxylate groups (–COO^−^). This band confirms the successful introduction of carboxyl functionalities via TEMPO-mediated oxidation [31].

While the FTIR analysis provides qualitative evidence of oxidation, the extent of carboxylation was quantified using conductometric titration, which indicated carboxylate content (millimoles of carboxylate per gram of dry fiber) equivalent to 0.85 ± 0.08 and 1.00 ± 0.05 from MNFC-UB and MNFC-B, respectively. As noted, under identical oxidation conditions, MNFC-B exhibited a higher carboxylate content compared to MNFC-UB. This enhancement is attributed to the increased accessibility of primary hydroxyl groups for oxidation as a result of lignin and hemicellulose removal. Similar observations have been reported for never-dried sulfite softwood pulp [58] and pineapple leaf fiber [6].

The phenolic groups in lignin react readily with oxidizing agents such as ClO_2_ and NaClO. As a result, some oxidant was consumed in non-selective side reactions, reducing its availability for selective cellulose oxidation [59,60]. In the current study, a dosage of 2.5 mmol NaClO per gram of fiber was applied. This is more than double the 1.2 mmol/g used by [6] for PALF cellulose oxidation, resulting in carboxylate levels approximately four times higher for the unbleached MNFCs and three times higher for the bleached ones.

Modulating the degree of oxidation is essential for tailoring nanocellulose properties to specific applications. Higher oxidation levels facilitate mechanical disintegration and improve colloidal stability, though excessive oxidation can weaken the structural stability of the cellulose and negatively impact mechanical performance [36,50]. We note environmental concerns about the TEMPO oxidation process; therefore, efforts have been made to improve the environmental sustainability of the process by using less NaClO, recirculating reactants, and evaluating alternative oxidizing agents, which have also been proposed, opening the potential to reduce environmental impacts [61].

Interestingly, pineapple-derived fibers obtained via alkaline (soda) pulping and oxidized under the same NaClO conditions have been shown to yield lower degrees of oxidation. This gives evidence about the effectiveness of organosolv pulping, which produces fibers with reduced lignin and hemicellulose content, thus improving oxidation efficiency [42,62]. Zeta potential reflects surface charge and colloidal stability of micro-/nanofibrillated cellulose suspensions. The measured values for MNFC-UB and MNFC-B corresponded to −40.7 ± 4.9 mV and −52.6 ± 6.1 mV, indicating good colloidal stability.

The higher degree of oxidation correlated with a more negative zeta potential, as increased surface carboxylation enhances the electrostatic repulsion between micro-/nanofibers. Since all the measurements were conducted at neutral pH, the –COOH groups on the cellulose surface were expected to be deprotonated into –COO^−^ (carboxylate anions). This deprotonation is consistent with the known pKa range (2.8–3.7) of C_6_-carboxyl groups in polyuronic acids [63]. Consequently, MNFCs derived from bleached fibers, which exhibit higher carboxylate content, also showed a more negative surface charge, in agreement with the oxidation degree results discussed previously.

As noted earlier, MNFC-B exhibited smaller diameters than their unbleached counterparts, which is consistent with their higher degree of oxidation. The increased surface carboxylation facilitates water retention and promotes higher nanofibrillation yield, resulting in finer and better-individualized fibrils.

X-ray diffraction (XRD) patterns were acquired for PALF and MNFCs (Figure 7b). The XRD pattern of the pineapple leaf fibers (PALF) showed characteristic features of cellulose I, the dominant allomorph in most native plant celluloses. A broad peak was observed at 14.9° (2θ), corresponding to the overlapping of the reflections ($\bar{11}0$) and (110) planes. A sharper peak at 22.5° (2θ) is assigned to the (200) plane, and the latter is frequently used to calculate the crystallinity index (CI) according to the Segal method, and a lower intensity peak is found at 34.4° (2θ), associated with the (004) plane [64,65,66].

For both nanocellulose samples, the peak at 22.5° appeared more intense, reflecting a higher proportion of crystalline cellulose. This increase resulted from the progressive removal of lignin and hemicellulose during purification, oxidation, and microfluidization. The bleached nanocellulose exhibited the most intense (200) peak, which is consistent with its higher crystallinity: 71% (PALF), 80% (MNFC-UB), and 85% (MNFC-B).

These results demonstrate a progressive increase in crystalline content from the precursor biomass to MNFCs, with the bleached micro-/nanocellulose showing the highest crystallinity index. The values obtained are higher than those reported in prior studies on pineapple leaf fiber MNFC obtained via soda pulping [6], underscoring the effectiveness of organosolv pulping in producing fibers with enhanced cellulose crystallinity. Increased crystallinity is beneficial for applications that demand high mechanical strength and structural integrity, such as nanocomposites and reinforced polymer matrices [67].

Further examination of the diffractograms revealed that the broad peak at 14.9° in PALF split into two distinct peaks in the MNFC samples: one around 13–14° and another near 16°. The latter is assigned to the (110) plane of cellulose I, associated with the formation of more ordered crystalline domains [64,65].

No peaks associated with cellulose II (typically at 12.2°, 20.2°, and 21.8°) were observed, confirming that the native cellulose I structure was retained. This peak-splitting phenomenon was not reported in prior soda-based pulping studies for pineapple MNFCs [6,31,68]. More extensive studies at the peak splitting at ~14.9° are required, since this is expected for highly purified samples [65] or sources like cotton [69].

### 3.4. Thermal Stability

**Figure 8:**
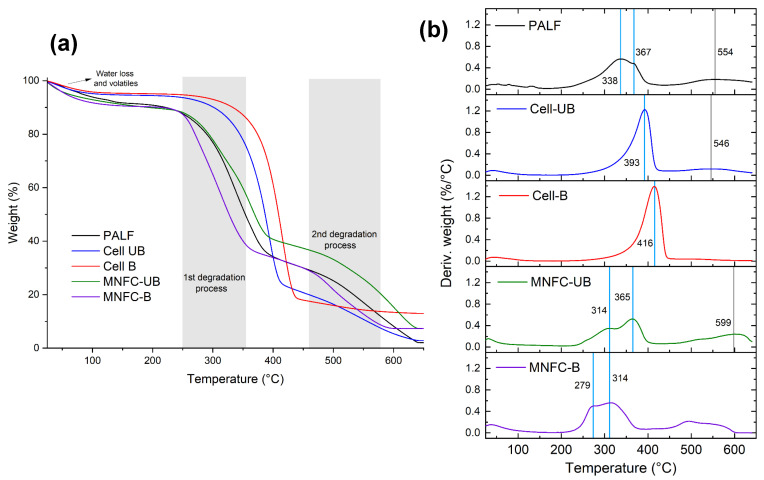
(**a**) Thermogravimetric (TGA) and (**b**) derivative thermogravimetric (DTG) curves for PALF, cellulose (Cell-UB and Cell-B), and micro-/nanofibrillated cellulose (MNFC-UB and MNFC-B).

To assess the thermal degradation behavior and stability of PALF, cellulosic fibers, and micro-/nanofibrillated cellulose samples, a thermogravimetric analysis (TGA) was conducted. Figure 8 presents the TGA curves and derivative thermogravimetric (DTG) profiles. The degradation of PALF shows an initial weight loss stage associated with water loss and release of volatiles, followed by two main degradation processes, which indicate a heterogeneous composition of the material. The first process corresponds to hemicellulose degradation (200–350 °C) and cellulose (350–480 °C). In this case, it is observed that there is a main peak with a shoulder, which can represent the overlapping of both degradation processes. At higher temperatures (>480 °C), the decomposition of the lignin occurs; in this case, the degradation was around 550 °C [70]. The residual material above 600 °C is associated with the existence of inorganic components, known as ashes. For PALF, the presence of silica particles has been reported [71], which contributes to this residual fraction. A summary of the key thermal processes is presented in Table 3.

The thermal analyses are relevant to the possible application of micro-/nanocellulose for applications such as polymer reinforcement, packaging, and thermal insulation. The balance between thermal stability and chemical functionality must be considered when designing cellulose-based materials for specific industrial uses.

For the fibers, both the unbleached and bleached samples exhibited an increase in the onset temperature of the first degradation stage. As noted previously, this degradation stage is attributed to thermal degradation of hemicellulose and cellulose. The removal of hemicellulose and lignin during the bleaching process causes crystallinity increase, which is confirmed by X-ray diffraction analysis. This enhanced crystallinity contributes to the improvement of thermal stability. Notably, the unbleached fibers showed a second degradation stage above 500 °C, which may be attributed to residual lignin, since lignin is known to degrade at elevated temperatures (>480 °C). For the bleached fibers, this is not shown, indicating the effective removal of lignin.

The thermal degradation of MNFCs occurred in three stages. The first stage occurred by the desorption of water, and then two overlapping peaks are shown, resulting from the surface oxidation of cellulose and leading to a heterogeneous material. Compared to PALF and the corresponding cellulosic fibers, the nanofibers degraded at lower temperatures. This reduced thermal stability is associated with TEMPO-mediated oxidation, which introduces sodium anhydroglucuronate units, an observation confirmed by FTIR. The first peak corresponds to the degradation of these oxidized units, associated with the decarboxylation process of the oxidized material, aromatization, and sodium carbonate formation, and the second peak is related to the remaining unmodified cellulose, when the rupture of the glycosidic structure occurs, along with aromatization and sodium carbonate formation [72,73].

A decrease in thermal stability has been previously reported for MNFC and is associated with the presence of carboxylate groups on the cellulose; these groups are thermally less stable [6,30]. The reduced thermal stability of MNFC could represent limitations for certain applications, such as in composite materials that require high processing temperatures. Several strategies have been reported to improve thermal stability and expand applications for this nanopolysaccharide.

Several strategies have been proposed to address the thermal stability issue of MNFCs, for example, carboxylate group modification by methylation with trimethylsilyl diazomethane (TMSCHN_2_) [72,74]. Amidation of TEMPO oxidized nanofibers, either using an amine-terminated polyethylene glycol (PEG-NH_2_) to form carboxylate ammonium salt-type structures [75], or modification with octadecylamine (ODA), forms an amide bond, which also increases the hydrophobicity of the nanofibers as a result of the incorporation of long alkyl chains [76]. The generation of silane-modified nanofibers with 3-aminopropyltrimethoxysilane (APS) and their blending with a polymer matrix such as PLA has also been proposed [77].

Other strategies are the exchange of counterions of the carboxylate group, replacing Na^+^ ions with multivalent cations such as Ca^+2^ and Mg^+2^ [72], and the inorganic coating of cellulose nanofibers, such as silica coating, which acts as a barrier against degradation [78].

## 4. Conclusions

The present study demonstrates the technical feasibility of employing a sequential process, applied here for the first time, that includes organosolv pulping to process pineapple leaf fibers (PALFs). The extracted PALF were converted into TEMPO-oxidized micro-/nanofibrillated cellulose (MNFC) through TEMPO-mediated oxidation followed by microfluidization, achieving nanofibrillation yields of 73% (bleached) and 68% (unbleached). The resulting MNFC, with carboxylate contents up to 1.00 mmol/g, exhibited fibril widths of 12–15 nm and zeta potential values below –40 mV. The bleached MNFC (MNFC-B) showed a crystallinity index of 85%.

Morphological analyses confirmed the progressive transformation from compact fiber bundles in raw PALF to partially disaggregated micrometer-sized cellulosic fibers after organosolv pulping and bleaching, and finally to nanofibrils with widths below 25 nm after TEMPO oxidation and microfluidization. By employing complementary characterization techniques—FQA, SEM, TEM, AFM, FTIR, XRD, and TGA—the conversion of precursor cellulosic fibers into micro-/nanofibrils was evidenced, while also indicating the presence of a residual non-nanofibrillated fraction.

The MNFC produced formed fibrillar networks with nano- and micro-scale widths and favorable structural and colloidal properties. Their available carboxylate groups, high surface charge, crystallinity, and aspect ratio make them suitable as processing matrices, nanopapers, biomedical materials, and environmental applications.

Overall, this work identifies pineapple leaf fibers as a viable biomass resource for the development of advanced nanomaterials, opening opportunities for PALF valorization. Future research should focus on recovering and characterizing process by-products, developing application pathways, and evaluating scalability through techno-economic and environmental analyses.

## Figures and Tables

**Table 1:** Results of Fiber Quality Analysis (FQA) of cellulosic fibers extracted from PALF and conducted in accordance with ISO 16065.

	Unbleached Fibers	Bleached Fibers
Mean length [mm] ^1^		
Arithmetic, LN	0.47 ± 0.02	0.33 ± 0.01
Length weighted, LW	0.78 ± 0.05	0.47 ± 0.02
Weight weighted, LZ	1.33 ± 0.09	0.78 ± 0.02
Mean width [µm]		
Arithmetic, W	12.25 ± 0.07	12.2 ± 0.4
Percent fines [%]		
Arithmetic	66 ± 1	88.4 ± 0.3
Length weighted	33 ± 2	72 ± 1
Mean curl index		
Arithmetic, CIn	0.119 ± 0.003	0.135 ± 0.004
Length weighted, CIw	0.135 ± 0.007	0.139 ± 0.006
Mean kink index		
Kink index [1/mm], KI	2.46 ± 0.01	2.59 ± 0.06
Total kink angle [°]	42.4 ± 0.9	40.2 ± 0.5
Kinks per mm [1/mm]	1.225 ± 0.007	1.23 ± 0.04

^1^ ISO 16065 defines fibers as having lengths larger than 0.20 mm, and fines are considered the particles in the range of 0.07 and 0.20 mm.

**Table 2:** Comparative morphological evidence of PALF transformation into micro-/nanofibrillated cellulose (MNFC).

Processing Stage	Technique(s)	Observed Morphology	Dimensions
Raw pineapple leaf fibers (PALFs)	Visual, confocal fluorescence microscopy, SEM	Bundled fibrous tissues embedded in parenchyma; heterogeneous structure	Width: Variable dimensions in micrometer range. Length: variable dimensions in cm range. Mean width: 159 ± 2 µm (SEM)
Cellulosic fibers: Unbleached (Cell-UB)	FQA, SEM	Partially disaggregated fibers; residual lignin/hemicellulose visible	Mean length, FQA: 0.47 ± 0.02 mm Mean width: 12.25 ± 0.07 µm (FQA), 10 ± 2 µm (SEM)
Cellulosic fibers: Bleached (Cell-B)	FQA, SEM	Cleaner, smoother fibers; more homogeneous appearance	Mean length, FQA: 0.33 ± 0.01 mm Mean width 12.2 ± 0.4 µm (FQA), 9 ± 2 µm (SEM)
MNFC from Cell-UB (MNFC-UB)	TEM, AFM	Nanofibrillar network with some agglomeration	Mean width: 15 ± 4 nm (TEM); 22 ± 4 nm (AFM)
MNFC from Cell-B (MNFC-B)	TEM, AFM	Nanofibrillar network, thinner nanofibrils with improved dispersion	Mean width: 12 ± 3 nm (TEM); 20 ± 4 nm (AFM)

**Table 3:** Thermogravimetric data of PALF, cellulosic fibers, and micro-/nanofibrillated cellulose.

Material	PALF	Unbleached Cellulosic Fibers (Cell-UB)	Bleached Cellulosic Fibers (Cell-B)	MNFC-UB	MNFC-B
T_onset_ [°C]	256	315	359	262	252
	496	470	-	486	470
T_max_ [°C]	54	42	45	32	38
	338/367	393	416	314/365	279/314
	554	546		599	495
Residue at 650 °C [wt%]	1.8	2.8	13.0	7.3	7.3

## Data Availability

The original contributions presented in this study are included in the article. Further inquiries can be directed to the corresponding author (s).
